# An Opposite Pattern to the Conventional Thermal Hypothesis: Temperature-Dependent Variation in Coloration of Adults of *Saccharosydne procerus* (Homoptera: Delphacidae)

**DOI:** 10.1371/journal.pone.0128859

**Published:** 2015-05-29

**Authors:** Haichen Yin, Muhammad Shakeel, Jing Kuang, Jianhong Li

**Affiliations:** 1 College of Plant Science & Technology, Huazhong Agricultural University, Wuhan, China; 2 Wuhan Vegetable Research Institute, Wuhan, China; University of Lausanne, SWITZERLAND

## Abstract

Melanism is a common polymorphism in many insect species that also influences immune function. According to the thermal melanin hypothesis, ectothermic individuals from cooler environments have darker cuticles and higher polyphenol oxidase (PO) levels, which represent a better immunocompetence. In this study, the links among environmental temperature, melanism, and PO activity of *Saccharosydne procerus* (Matsumura) were examined. Most *S*. *procerus* have a black spot on their forewings at high temperatures in the field and in the laboratory. In PO activity assay, a positive association between PO level and temperature was found. Our results showed that a diversification of melanism occurred under different temperatures and that melanism in *S*. *procerus* presented an opposite pattern to the one proposed by the thermal hypothesis.

## Introduction

Melanism is a polymorphism in the same species or in related species in which dark color pigments develop in individuals [1]. Melanism is a common visible trait of many insect species [2]. The most widely known example of melanism is the peppered moth (*Biston betularia* Linn). During the nineteenth century, the frequency of melanic phenotypes increased rapidly and was considered to be a response to environmental change [3].

Color polymorphism not only reflects gene variation but also the plasticity of the environment [4], and the polymorphism is a strategy for insects to adapt to alterations in the environment [5]. Hence, environmental factors including background color, photoperiod, population density, and temperature affect the selection of body color. A variety of studies examined the relationship between melanism and the environment. For example, the dorsal surface of the sand-burrowing beetle (*Chaerodes trachyscelides*) closely matched the color of the sand in their environment [6], and the Egyptian cotton leaf worm (*Spodoptera littoralis*) developed darker cuticles when reared under high density [7]. Female *Tetranychus* spider mites became conspicuously orange when exposed to short-day and low-temperature conditions [8], and the cuticular melanization of the ground cricket *Allonemobius* was affected by the thermal environment [9].

Because insects are ectothermic, melanism may affect the absorption of heat. The thermal hypothesis states that dark individuals absorb heat faster under low-temperature conditions compared with individuals lighter in color [10]. Hence, body color variation results in different body temperatures, which affect many fundamental physiological functions and life history traits including reproduction, survival rate, and resistance to disease [10–13].

Moreover, cuticular pigmentation requires the oxidation of cuticular catechols to ortho-quinones by polyphenol oxidase (PO) [9]. There are two types of PO in insects, laccase and tyrosinase PO, respectively [14]. They have different functions, laccase PO facilitates cuticle darkening while tyrosinase PO facilitates melanin-based immunity [15, 16]. Thus, PO enzyme is involved in many physiological roles, including pigmentation, immune response, and wound healing [17–20]. At the infection or injury site, the cuticle typically becomes dark because melanization shares the same biochemical pathway with synthesis of immune-related melanin [13, 16, 21].

The melanin-based immune system, in a number of insect species, is regulated by environmental factors such as the thermal environment because temperature influences the host-pathogen interaction by regulating pathogen growth and host disease resistance [22,23]. A host will invest more in immune function when exposed to a greater risk of infection by a pathogen [24], as a result this will influence body color.

**Fig 1:**
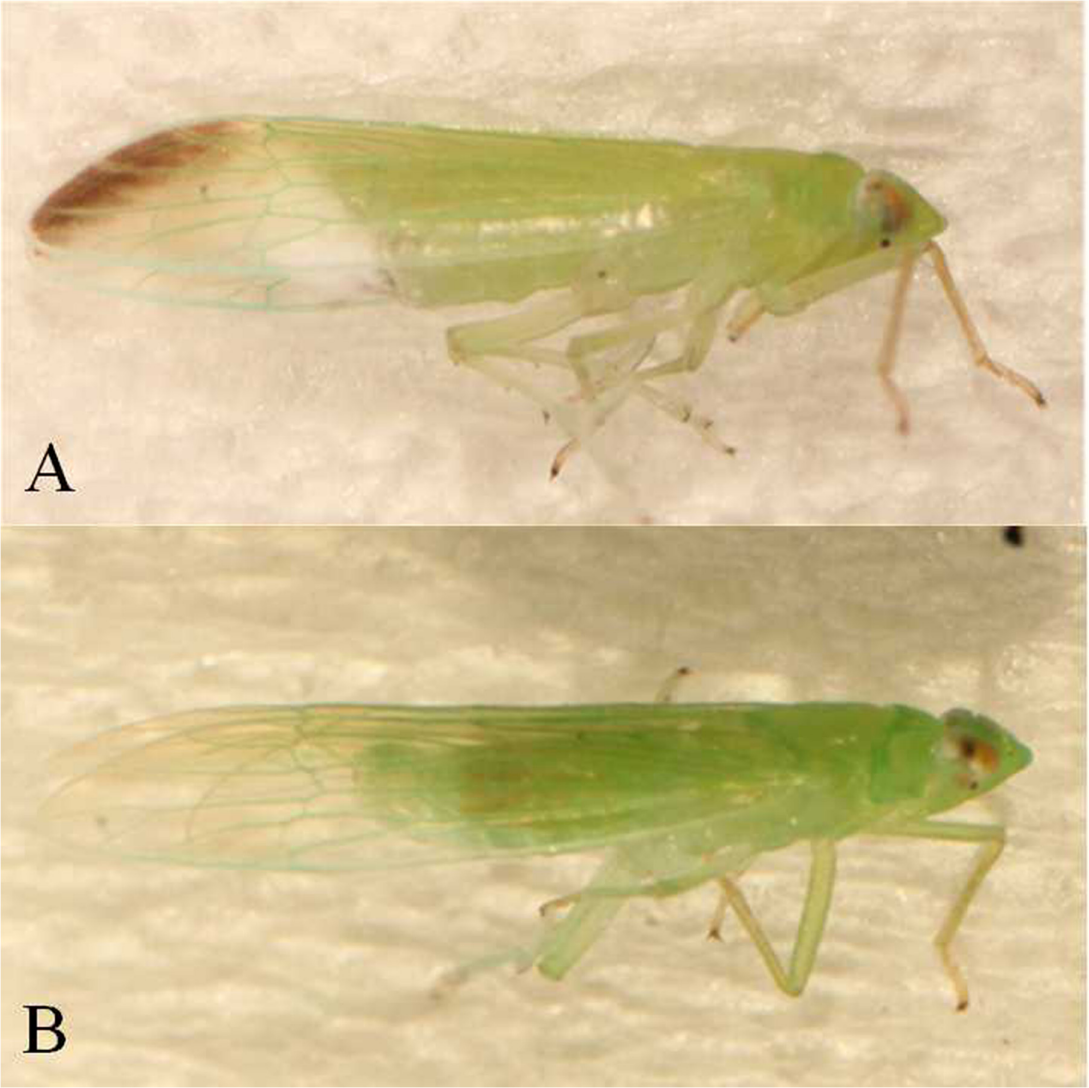
The melanic and non-melanic morphs of *Saccharosydne procerus*. A. Melanic morph and B. Non-melanic morph.

Although much information is available about melanism in insects, little is known about the melanism of pests of aquatic vegetables, particularly water bamboo. We examined the relationship between the environment and melanism in the green slender plant hopper (*Saccharosydne procerus* Matsumura), which is the primary pest of water bamboo (*Zizania latifolia*) and rice (*Oryza sativa*) in East Asian countries, including China and Vietnam. The species has two morph forms. The melanic morph has a black spot on the terminus of the forewing (Fig 1A), whereas the wing is transparent in the other morph (Fig 1B). The black spot appears after emergence, while the melanism of this species don’t change in adult stages. In this study, we examined the effect of environmental temperature on the proportion of melanic individuals and the activity levels of PO at the end of nymph stage, as we consider it to be a critical period for melanism. If the melanism of green slender plant hoppers was triggered by temperature, the hypothesis was that temperature will influence the proportion of melanic morphs and PO activity.

## Materials and Methods

### Ethics Statement

No specific permits were required for the described field studies. The field studies did not involve endangered or protected species.

### Rearing of *S*. *procerus*

The *S*. *procerus* used in present study were reared on water bamboo, which was grown in the greenhouse of Huazhong Agriculture University, Wuhan (N30°28’ and E114°21’), China. The greenhouse was only covered by gauze net to provide living conditions similar to the natural habitat. The plant hoppers were not exposed to any pesticides, and therefore, they did not face any selection pressure from pesticides.

### Field investigation

The proportion of melanic morphs was investigated at five major water bamboo producing areas: Wuhan (N30°28’ and E114°21’), Anqing (N30°35’ and E117°07’), Suzhou (N31°19’ and E120°37’), Changsha (N28°13’ and E112°56’), and Jinhua (N29°04’ and E119°38’). In Wuhan, the melanism proportion was recorded once a month from June to October in 2012 because the population density was sufficient in the field during this period. At the other locations, the investigations were conducted from July to September 2012. In each area, five plots were selected in a rectangular field (the area was approximately 30 m × 40 m), with one in the center and the other four in the corners. Leaves with adults were cut off and stored in plastic bags. In total, 600 individuals (120 individuals from each plot) were collected in each area. The proportion of melanic morphs was recorded in the field.

To compare the relationship between environmental temperature and melanism, temperature was recorded three times a day (08:00, 14:00, and 20:00) in Wuhan by a thermometer that was in the experimental field. At the other four locations, the weather information was downloaded from the Internet (Table 1). According to a previous survey, one generation of green slender plant hoppers was approximately 30 days [25]; therefore, the mean temperature of the 30 days prior to the investigation date was calculated.

**Table 1:** Descriptions of field locations.

Area	Latitude and Longitude	Date	Mean Temperature
Wuhan	N30°28’ and E114°21’	2012.6.8	23.19°C (2012.5.10–2012.6.8)
		2012.7.15	29.19°C (2012.6.16–2012.7.15)
		2012.8.16	30.34°C (2012.7.18–2012.8.16)
		2012.9.15	26.27°C (2012.8.17–2012.9.15)
		2012.10.15	21.72°C (2012.9.16–2012.10.15)
Anqing	N30°35’ and E117°07’	2012.9.14	26.58°C (2012.8.16–2012.9.14)
Suzhou	N31°19’ and E120°37’	2012.7.24	29.52°C (2012.6.25–2012.7.24)
Changsha	N28°13’ and E112°56’	2012.6.19	24.75°C (2012.5.21–2012.6.19)
Jinhua	N29°04’ and E119°38’	2012.7.1	25.63°C (2012.6.2–2012.7.1)

### The effect of temperature on melanism

The eggs of green slender plant hoppers were collected from the field by cutting the water bamboo leaves with spawning marks in Wuhan. These eggs were placed in an artificial climate box at 26°C and L: D 16:8 h photoperiod. The nymphs were divided evenly and were maintained at seven temperature treatments (32°C, 30°C, 28°C, 26°C, 24°C, 22°C, and 18°C; L: D 16:8 h). For each treatment, the plant hoppers were fed with water bamboo leaf wrapped with wet cotton at the bottom in a glass tube (14 cm × 1.5 cm). Each tube contained one water bamboo leaf and 5 nymphs, and 100 individuals were used in each treatment. The leaves were changed daily. If mortality of nymphs occurred during the experiment, new first instar nymphs were added to keep the population density constant. After emergence, the adults were placed on a tissue paper to serve as a background, and the proportion of melanic morphs was recorded.

### Circulating PO activity assay

The circulating PO activity was compared in nymphs near emergence (two to three days after the fifth instar, according to a pre-experiment [23]) that were reared from the first instar under three temperature treatments (22°C, 26°C, and 30°C). To extract PO, 20 nymphs were homogenized in 0.5 ml of 0.1 mol/l pre-cooled PBS buffer (pH = 7) with a glass homogenizer and then were centrifuged at 4°C and 10,000 r/min for 10 min. The supernatant was carefully transferred into a precooled 1.5 ml eppendorf tube. The substrate used was catechol. Catechol (0.25 ml of 0.01 mol/l) was added to 0.25 ml of 0.1 mol/l PBS buffer (pH = 6.2) and incubated at 30°C for 10 min. The insect homogenate (0.4 ml) was added to the mixture. The change in OD was recorded at 420 nm at 10 min by a spectrophotometer (UV-1800, Shimadzu Suzhou Instruments Mfg. Co., Ltd.). In the present study, one PO unit was defined as the amount of enzyme that increased the OD by 0.01 per min, and the enzyme activity was expressed as PO units per g sample [26].

### Statistical analyses

Chi-squared test was employed to analyze (1) the variation of melanism in the field (2) the effect of temperature on melanization. We used one-way ANOVAs and pair-wise assessment was done using the Multiple Range Test method to analyze the PO activity of the two morphs. All analyses were conducted by SPSS Statistics 17.0.

## Results

### Field investigation

**Fig 2:**
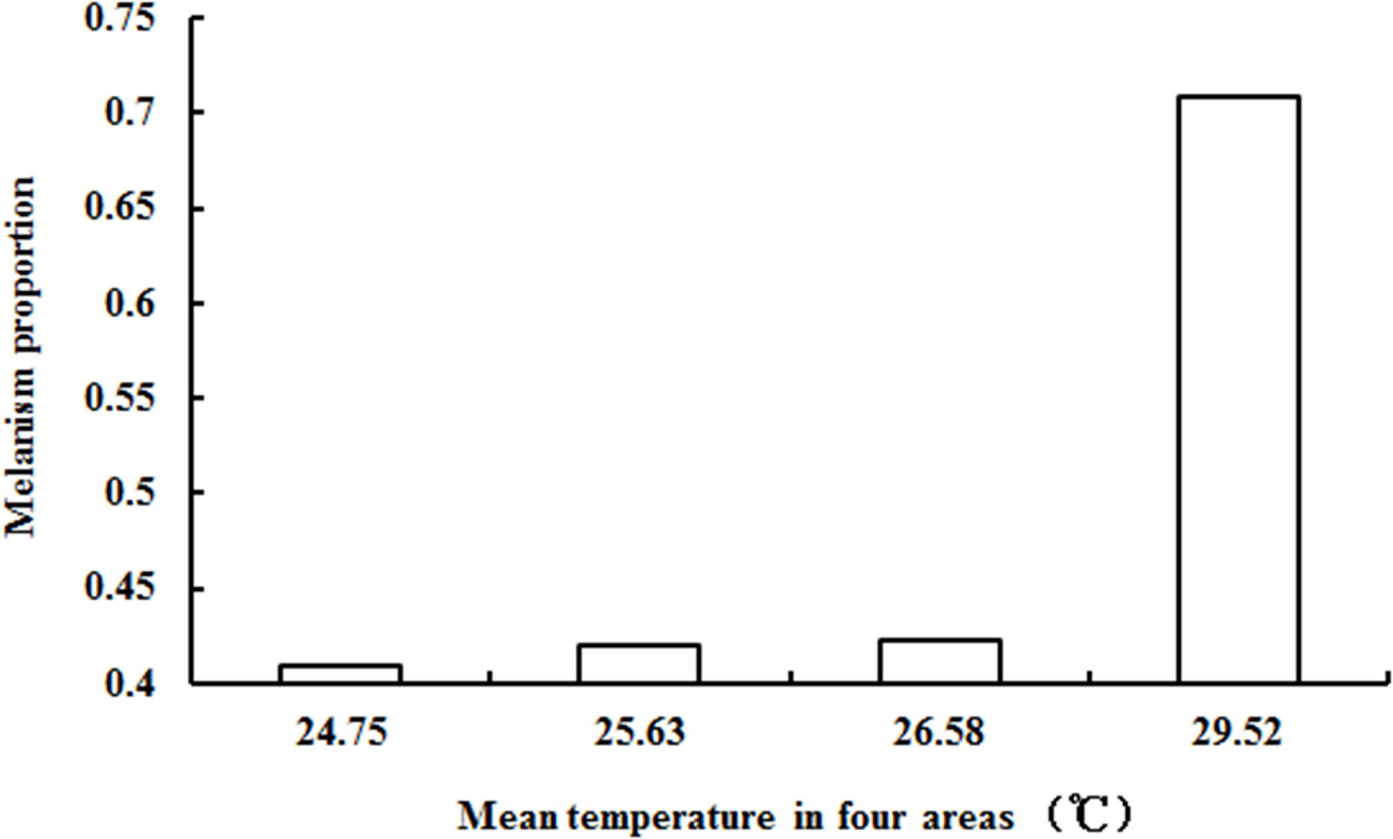
The variation in the proportion of melanism in adults of the *Saccharosydne procerus* in the primary water bamboo producing areas. The X axis represents the mean temperature of the 30 d before the investigation in the primary water bamboo producing areas: 24.75°C (Changsha, 2012.5.21–2012.6.19), 25.63°C (Jinhua, 2012.6.2–2012.7.1), 26.58°C (Anqing, 2012.8.16–2012.9.14), 29.52°C (Suzhou, 2012.6.25–2012.7.24). The mean temperature was the highest in Suzhou and the proportion of melanism was also significantly higher than that in the other three areas.

The field investigation found melanism in *S*. *procerus* in all four primary water bamboo producing areas; however, the proportion of melanic morphs was different in the four areas. The proportion of melanic morphs in Suzhou was significantly higher than the proportions found in Anqing (Pearson Chi-square = 99.190, df = 1, *P<*0.001), Jinhua (Pearson Chi-square = 101.434, df = 1, *P<*0.001) and Changsha (Pearson Chi-square = 99.190, df = 1, *P<*0.001). And the mean temperature of Suzhou was the highest when the investigation was conducted (Fig 2).

**Fig 3:**
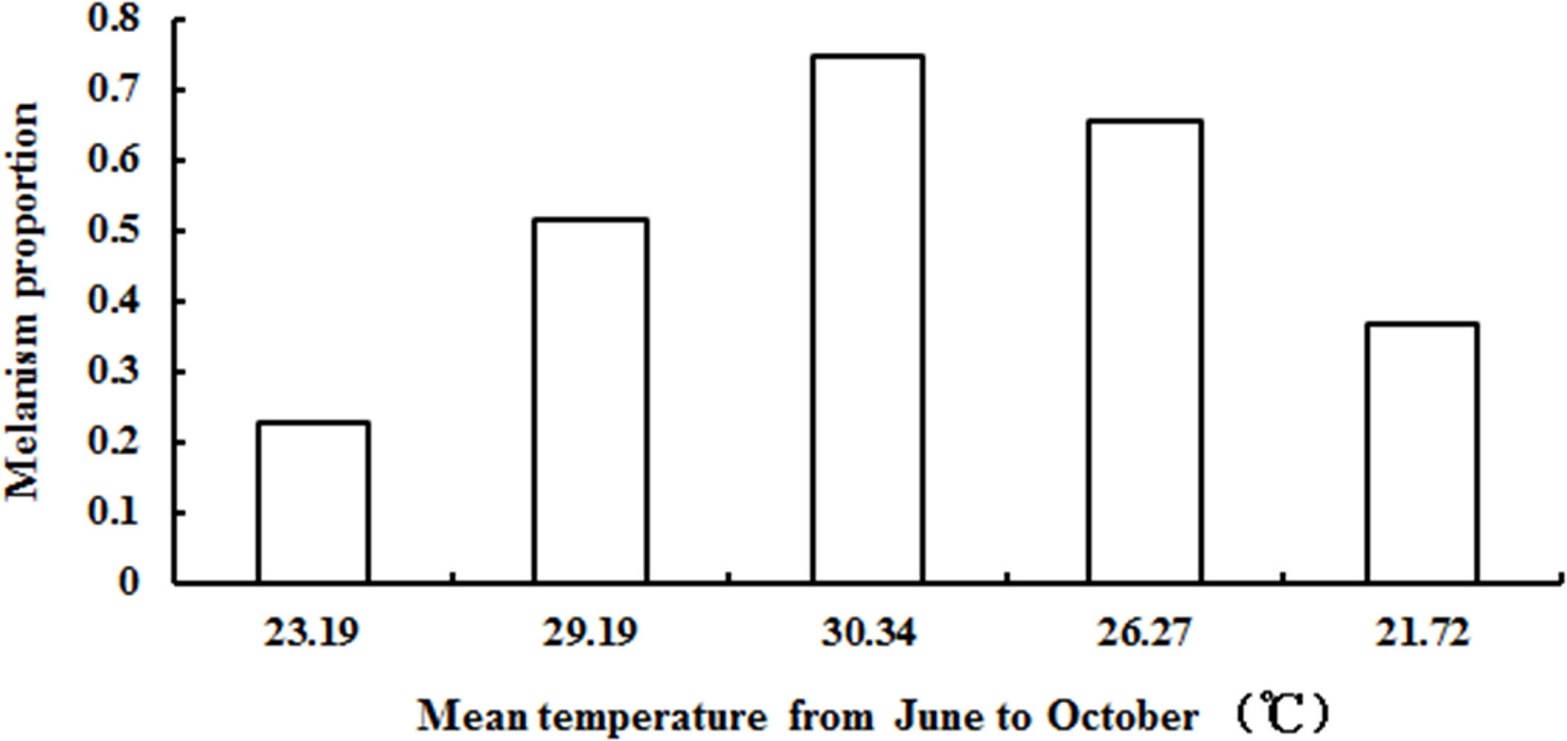
The variation in proportion of melanism in adults of *Saccharosydne procerus* in Wuhan from June to October 2012. The x-axis represents the mean temperature of the 30 d before the investigation in Wuhan: 23.19°C (2012.5.10–2012.6.8), 29.19°C (2012.6.16–2012.7.15), 30.34°C (2012.7.18–2012.8.16), 26.27°C (2012.8.17–2012.9.15), 21.72°C (2012.9.16–2012.10.15). The proportion of melanism and the mean temperature both reached the maximum level on 2012.8.16, indicating that the proportion of melanism changed with temperature.

In Wuhan, the proportion of melanism changed from June to October. The number of melanic morphs increased continually from June to August and then decreased from September to October. The melanism proportion was higher in August than that in June (Pearson Chi-square = 328.832, df = 1, *P<*0.001), July (Pearson Chi-square = 71.275, df = 1, *P<*0.001), September (Pearson Chi-square = 12.525, df = 1, *P<*0.001) and October (Pearson Chi-square = 175.812, df = 1, *P<*0.001). The mean temperature in the field was also highest in August 2012 (Fig 3).

### The effect of temperature on melanism

**Fig 4:**
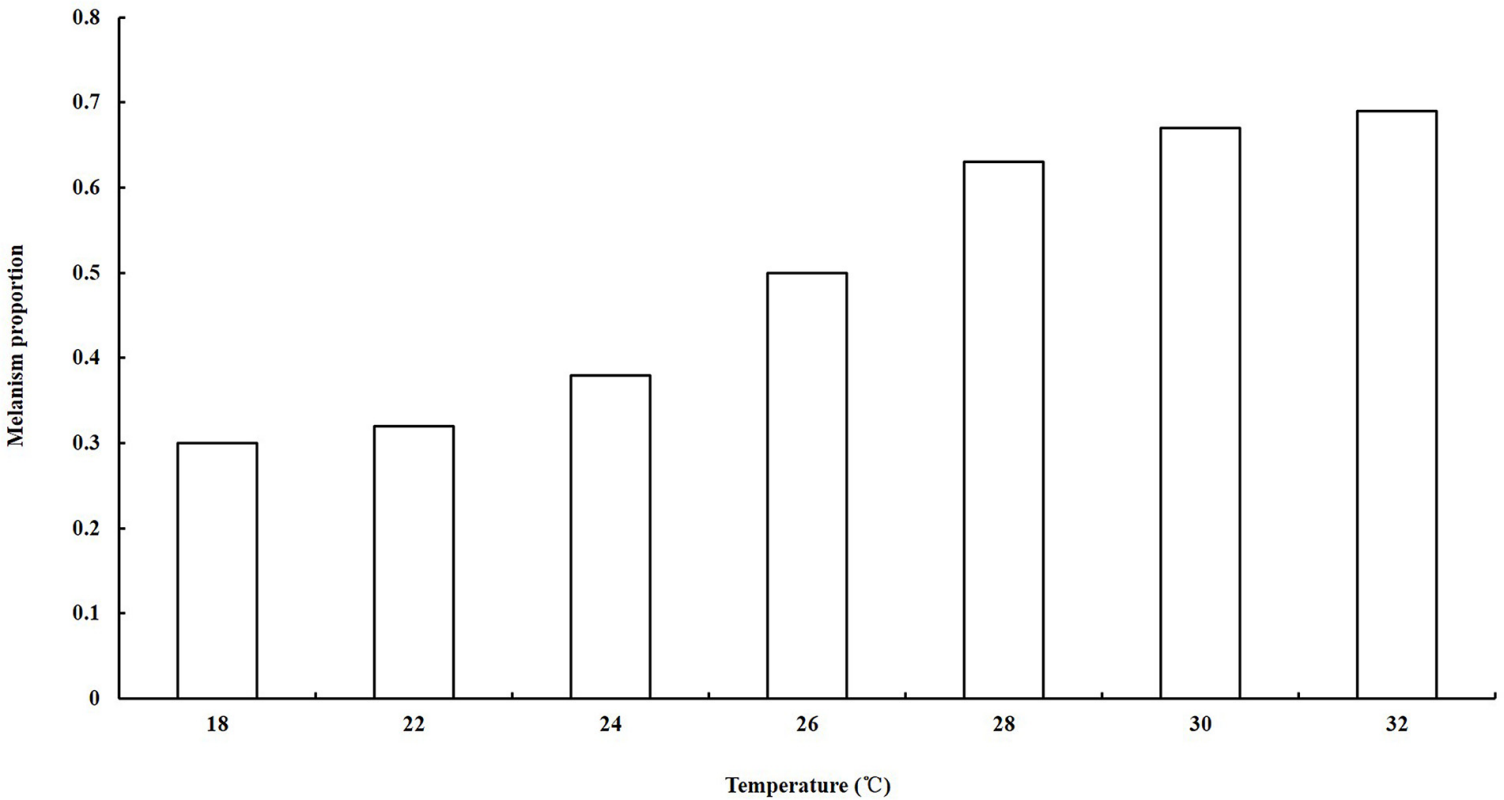
The proportion of melanic morphs at different temperatures. The proportion of melanic morphs increased with an increase in the temperature. The proportion of melanism at 30°C was significantly higher than that at 26°C, and the plant hoppers at 26°C showed a significantly higher proportion of melanism than those at 22°C. Our data demonstrated the relation between the proportion of melanism and temperature.

In the comparison of variation in body color and temperature, we inferred that environmental temperatures might influence the melanism of green slender plant hoppers. To verify this assumption, we compared the proportions of melanism at seven temperature treatments. The number of melanic morphs increased when the temperature increased. The proportion of melanism at 30°C was significantly higher than that at 26°C (Pearson Chi-square = 5.952, df = 1, *P =* 0.015), and the plant hoppers at 26°C showed a significantly higher proportion of melanism than those at 22°C (Pearson Chi-square = 6.697, df = 1, *P =* 0.01; Fig 4).

### Circulating PO activity assay

**Fig 5:**
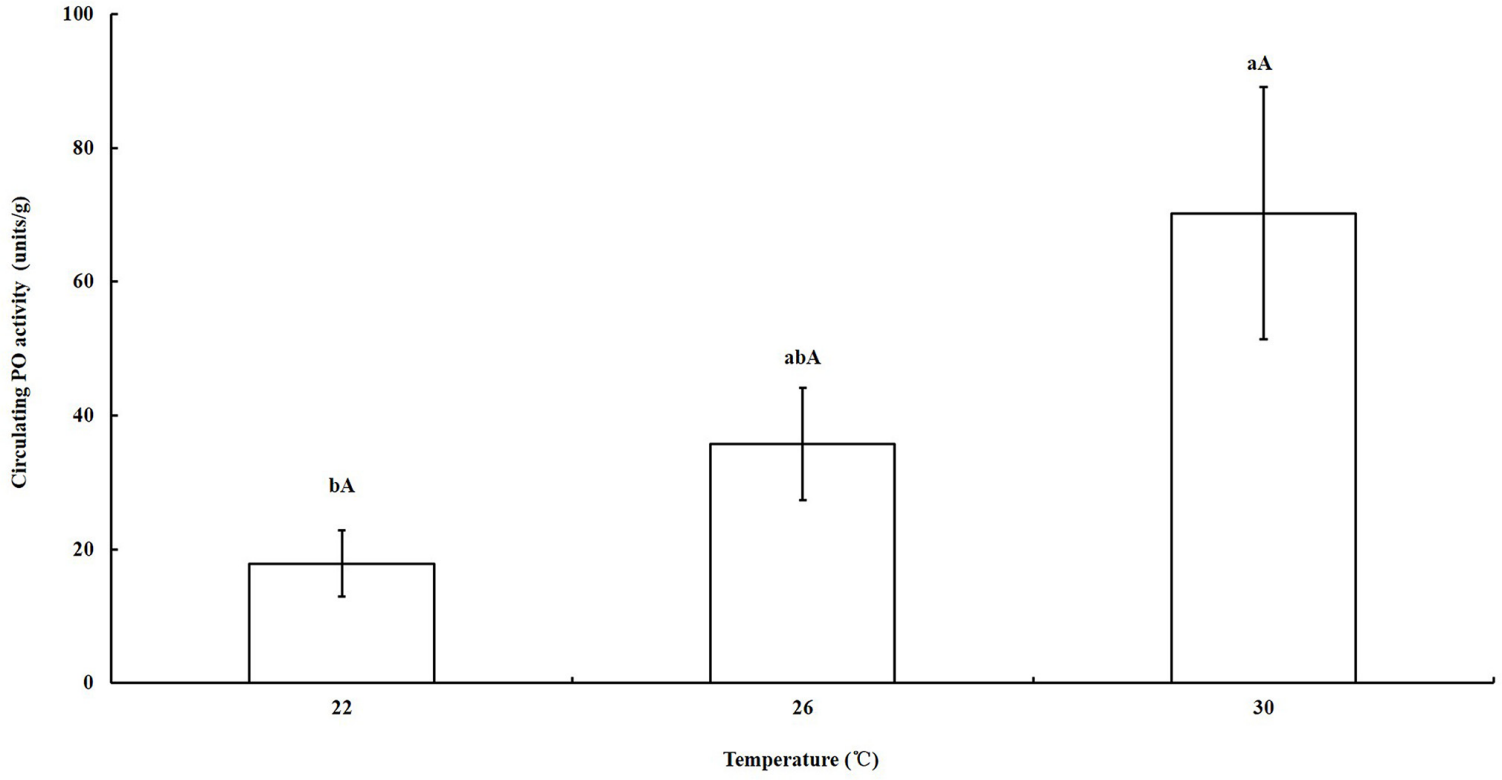
The circulating PO activity of nymphs near emergence reared at different temperatures. The activity of circulating PO (mean ± SE) was highest in nymphs when reared at 30°C and lowest at 26°C, which indicated that hot conditions triggered higher PO activity.

In the circulating PO activity assays, the PO activity of the nymphs reared from the first to fifth instar at 22°C, 26°C, and 30°C was compared. These temperatures were selected because of the significant differences in the proportions of melanism at these temperatures. The PO activity at 30°C was significantly higher than it was at 22°C (*P* = 0.035), whereas no significant differences in PO activity were found at 26°C compared with that at 30°C (*P* = 0.1709) and 22°C (*P* = 0.5764; Fig 5).

## Discussion

The present study was conducted to determine the associations among melanism of *S*. *procerus*, environmental temperature, and PO activity. Field investigations indicated that the environmental temperatures influenced the proportion of melanic morphs in field populations. The effect of temperature on melanism was then simulated in the laboratory. The results demonstrated that the number of melanic morphs increased with increased temperatures. To explain the mechanism of melanization, the PO level was measured, and the PO activity assay showed that the PO activity of the nymphs near emergence also increased with increased temperatures. In the nymph stage the melanism of *S*. *procerus* does not appear, while once emergence finishes the body color of *S*. *procerus* do not change any more. Thus, high levels of PO at the end of the nymph stage may lead to melanization. To date, we can find lots of studies on the relationship between PO and body color. For example, compared with non-melanic morphs melanic strain of the greater wax moth (*Galleria mellonella*) showed a higher PO level [27] and the melanization of Egyptian cotton leafworm (*Spodoptera littoralis*) positively correlated with its PO activity [28].

As discussed in the Introduction part, the PO has two types and they have different functions. Considering that melanism shares some same biochemical pathway with other physiological processes, the effect of ambient temperature on these processes such as immunity may indirectly shape the body color [16].

Interestingly our results are opposite to those predicted by the thermal hypothesis. The thermal hypothesis is widely accepted, and the expectation would be the proportion of melanic morphs would increase in cool conditions to absorb more heat from the environment [10]. However, the thermal hypothesis is not the only explanation for melanism because of the complicated mechanism to produce melanin. Hence, the effect of the thermal environment on insect melanism is complex. Although the thermal hypothesis is widely accepted, counter examples are found. In some cases, melanism was observed in warmer habitats, whereas individuals in cooler environments were lighter in color [29], and many melanic morphs were found in some tropical areas. These studies indicate that a universal explanation for melanism is not available, and that the evolution of body color is triggered by more than one ecological factor [30].

A previous study showed that individuals invest more in the immune system when they have a greater risk of infection [31]. As we discussed above when faced with infection or injury, the cuticle of insects typically becomes dark because of the higher PO activity and melanin synthesis [13, 21]. In the present study, we speculate that the body color of *S*. *procerus* was changed in the same way. High temperature may influence the host-pathogen interaction [22, 23], then PO level was changed because this enzymes was involved in immunity [32] and finally the variation in PO activity altered the body color.

Considering the body color variation under high temperature, we are interested in the effect of climate warming on the melanism. Organisms can adapt to this alteration through phenotypic plasticity including melanism which can deal with the increase in infectious diseases associated with global warming [33]. When faced with a high risk of infection, melanic morph usually have an advantage due to the better immunocompetence than that of lighter ones [32]. For example, melanic morphs of *Ephestia kuhuiella* were better able to inhibit the oviposition and larval development of parasitic wasps [34], and the melanic morphs of *Spodoptera exempta* showed a significantly higher resistance to baculovirus than non-melanic morphs[35]. Additionally, melanic *Tenebrio molitor* showed lower mortality when exposed to a generalist entomopathogenic fungus [36]. Therefore, we infer that the number of melanic *S*. *procerus* may increase if climate become warmer in future, due to the advantages of melanic morphs when faced with increased risk of infection caused by globle warming [37].

Melanin-based coloration can be costly to produce, maintain or wear [38], thus individuals need a trade-off between melanin-based coloration and other biological processes [39]. This may lead to the differences between the fitness of melanic morphs and that of non-melanic individuals [40]. Some hypotheses state that melanin-based coloration can be a criterion in mate choice under different environmental conditions because the choice based on body color may provide some direct or indirect benefits to adapt to altered environment [38]. For example, females of *Harmonia axyridis* prefer typical (succinea form) males to melanic ones in the spring generation due to the thermal disadvantages of melanism during summer [41]. While we infer that the mating behavior of *S*. *procerus* may be different from that of *H*. *axyridis* due to the opposite pattern to conventional thermal hypothesis, thus we think it is interesting to study the variation in the fitness of two morphs including mating behavior in the future.

In summary, we suggest that more melanic morphs and a high level of PO will appear in hot conditions. Although we did not simulate an immune challenge, PO activity, as an important component of the immune system, represented immunocompetence [42]. However in the present study, we did not distinguish between laccase or tyrosinase in the PO-assay. Thus we think it is necessary to explore this in the future. Moreover if the mean temperature increases in the future as a result of global climate change, we predict that more melanic morphs will appear in the field and that the prevalence of melanin-based immunity will increase. The results did not support the accepted thermal hypothesis and suggest it may be worthwhile to explore gene regulation and the effect of melanism on life history traits. Additionally, the photoperiod in this study was employed due to some previous studies of similar species [43, 44]. While it is not natural and may act as a superstimulus, however, it is difficult to find some previous studies about the effect of photoperiod on melanism of *S*. *Procerus*. Therefore, we think it is worthwhile to explore it in the future.

## References

[1] Clusella Trullas S, van Wyk JH, Spotila JR (2007) Thermal melanism in ectotherms. J Therm Biol 32: 235–245.

[2] True JR (2003) Insect melanism: the molecules matter. Trends Ecol Evol 18: 640–647.

[3] Grant B, Owen D, Clarke C (1996) Parallel rise and fall of melanic peppered moths in America and Britain. J Hered 87: 351–357.

[4] Karlsson M, Forsman A (2010) Is melanism in pygmy grasshoppers induced by crowding? Evol Ecol 24: 975–983.

[5] van’t Hof AE, Edmonds N, Dalíková M, Marec F, Saccheri IJ (2011) Industrial melanism in British peppered moths has a singular and recent mutational origin. Science 332: 958–960. 10.1126/science.1203043 21493823

[6] Harris A (1988) Cryptic colouration and melanism in the sand-burrowing beetle *Chaerodes trachyscelides* (Coleoptera: Tenebrionidae). J Roy Soc New Zeal 18: 333–339.

[7] Lee KP, Wilson K (2006) Melanism in a larval Lepidoptera: repeatability and heritability of a dynamic trait. Ecol Entomol 31: 196–205.

[8] Ito K, Fukuda T, Hayakawa H, Arakawa R, Saito Y (2013) Relationship between body color, feeding, and reproductive arrest under short-day development in *Tetranychus pueraricola* (Acari: Tetranychidae). Exp Appl Acarol 60: 471–477. 10.1007/s10493-013-9660-3 23420142

[9] Fedorka KM, Lee V, Winterhalter WE (2013) Thermal environment shapes cuticle melanism and melanin-based immunity in the ground cricket (*Allonemobius socius*). Evol Ecol 27: 521–531.

[10] Clusella-Trullas S, Terblanche J, Blackburn T, Chown S (2008) Testing the thermal melanism hypothesis: a macrophysiological approach. Funct Ecol 22: 232–238.

[11] Clusella-Trullas S, Wyk JH, Spotila JR (2009) Thermal benefits of melanism in cordylid lizards: a theoretical and field test. Ecology 90: 2297–2312. 19739391 10.1890/08-1502.1

[12] Jong P, Gussekloo S, Brakefield P (1996) Differences in thermal balance, body temperature and activity between non-melanic and melanic two-spot ladybird beetles (*Adalia bipunctata*) under controlled conditions. J Exp Biol 199: 2655–2666. 9320589 10.1242/jeb.199.12.2655

[13] Wilson K, Cotter SC, Reeson AF, Pell JK (2001) Melanism and disease resistance in insects. Ecol Lett 4: 637–649.

[14] Futahashi R, Tanaka K, Matsuura Y, Tanahashi M, Kikuchi Y, Fukatsua T (2011) Laccase2 is required for cuticular pigmentation in stinkbugs. Insect Biochem Mol Biol 41: 191–196. 10.1016/j.ibmb.2010.12.003 21167282

[15] Masuoka Y, Miyazaki S, Saiki R, Tsuchida T, Maekawa K (2013) High *Laccase2* expression is likely involved in the formation of specific cuticular structures during soldier differentiation of the termite Reticulitermes speratus. Arthropod Struct Dev 42: 469–475. 10.1016/j.asd.2013.08.003 24076334

[16] Kutch IC, Sevgili H, Wittman T, Fedorka KM (2014) Thermoregulatory strategy may shape immune investment in *Drosophila melanogaster* . J Exp Biol 217: 3664–3669. 10.1242/jeb.106294 25147243

[17] Bailey NW (2011) A test of the relationship between cuticular melanism and immune function in wild-caught Mormon crickets. Physiol Entomol 36: 155–164.

[18] Zufelato MS, Lourenço AP, Simões ZL, Jorge JA, Bitondi MM (2004) Phenoloxidase activity in (*Apis mellifera*) honey bee pupae, and ecdysteroid-dependent expression of the prophenoloxidase mRNA. Insect Biochem Mol Biol 34: 1257–1268. 15544939 10.1016/j.ibmb.2004.08.005

[19] Yu X-Q, Jiang H, Wang Y, Kanost MR (2003) Nonproteolytic serine proteinase homologs are involved in prophenoloxidase activation in the tobacco hornworm, (*Manduca sexta*). Insect Biochem Mol Biol 33: 197–208. 12535678 10.1016/s0965-1748(02)00191-1

[20] Christensen BM, Li J, Chen C-C, Nappi AJ (2005) Melanization immune responses in mosquito vectors. Trends Parasitol 21: 192–199. 15780842 10.1016/j.pt.2005.02.007

[21] Eleftherianos I, Revenis C (2010) Role and importance of phenoloxidase in insect hemostasis. J Innate Immun 3: 28–33. 10.1159/000321931 21051882

[22] Cotter S, Myatt J, Benskin C, Wilson K (2008) Selection for cuticular melanism reveals immune function and life history trade-offs in (*Spodoptera littoralis*). J Evol Biol 21: 1744–1754. 10.1111/j.1420-9101.2008.01587.x 18691239

[23] Catalán TP, Niemeyer HM, Kalergis AM, Bozinovic F (2012) Interplay between behavioural thermoregulation and immune response in mealworms. J Insect Physiol 58: 1450–1455. 10.1016/j.jinsphys.2012.08.011 22985859

[24] Mills SC (2012) Density-dependent prophylaxis in the coral-eating crown-of-thorns sea star, (*Acanthaster planci*). Coral reefs 31: 603–612.

[25] Kuang J (2012) A Preliminary Study on the green slender planthopper (*Saccharosydne procerus*) in Wuhan area and Insecticides selection Wuhan: Huazhong Agricultural University. 42 p.

[26] Benjamin ND, Montgomery M (1973) Polyphenol oxidase of Royal Ann cherries: purification and characterization. J Food Sci 38: 799–806.

[27] Dubovskiy IM, Whitten MMA, Kryukov VY, Yaroslavtseva ON, Grizanova EV, Greig C et al (2013) More than a colour change: insect melanism, disease resistance and fecundity. P Roy Soc B-Biol Sci, 280: 20130584 10.1098/rspb.2013.0584 23698007 PMC3774225

[28] Cotter SC, Kruuk LEB, Wilson K (2004) Costs of resistance: genetic correlations and potential trade‐offs in an insect immune System. J Evolution Biol, 17: 421–429. 15009275 10.1046/j.1420-9101.2003.00655.x

[29] Rajpurohit S, Parkash R, Ramniwas S (2008) Body melanization and its adaptive role in thermoregulation and tolerance against desiccating conditions in *Drosophilids* . Entomol Res 38: 49–60.

[30] Wittkopp PJ, Carroll SB, Kopp A (2003) Evolution in black and white: genetic control of pigment patterns in *Drosophila* . Trends Genet 19: 495–504. 12957543 10.1016/S0168-9525(03)00194-X

[31] Wilson K, Reeson AF (1998) Density-dependent prophylaxis: evidence from Lepidoptera-baculovirus interactions? Ecol Entomol 23: 100–101.

[32] Armitage SAO, Siva-Jothy M (2005) Immune function responds to selection for cuticular colour in (Tenebrio molitor). Heredity 94: 650–656. 15815710 10.1038/sj.hdy.6800675

[33] Roulin A (2014) Melanin-based colour polymorphism responding to climate change. Global change biol 20: 3344–3350. 10.1111/gcb.12594 24700793

[34] Verhoog M, Van Boven A, Brakefield P (1996) Melanic moths and the ability to encapsulate parasitoid eggs and larvae. Proc Neth Entomol Soc 7: 127–133.

[35] Reeson AF, Wilson K, Gunn A, Hails RS, Goulson D (1998) Baculovirus resistance in the noctuid (*Spodoptera exempta*) is phenotypically plastic and responds to population density. Proc R Soc Lond B 265: 1787–1791.

[36] Barnes AI, Siva-Jothy MT (2000) Density–dependent prophylaxis in the mealworm beetle *Tenebrio molitor* L.(Coleoptera: Tenebrionidae): cuticular melanization is an indicator of investment in immunity. Proc R Soc Lond B 267: 177–182. 10.1098/rspb.2000.0984 PMC1690519 10687824

[37] Harvell D, Altizer S, Cattadori IM, Harrington L, Weil E (2009) Climate change and wildlife diseases: when does the host matter the most? Ecology, 90: 912–920. 19449685 10.1890/08-0616.1

[38] Roulin A (2015) Condition-dependence, pleiotropy and the handicap principle of sexual selection in melanin-based colouration. Biol Rev, 10.1111/brv.12171 25631160

[39] Sheldon BC, Verhulst S (1996) Ecological immunology: costly parasite defences and trade-offs in evolutionary ecology. Trends Ecol Evol 11: 317–321. 21237861 10.1016/0169-5347(96)10039-2

[40] Creed E, Lees D, Bulmer M (1980) Pre-adult viability differences of melanic *Biston betularia* (L.) (Lepidoptera). Biol J Linn Soc 13: 251–262.

[41] Wang S, Michaud JP, Tan XL, Murray L, Zhang F (2013) Melanism in a Chinese Population of Harmonia axyridis (Coleoptera: Coccinellidae): A Criterion for Male Investment with Pleiotropic Effects on Behavior and Fertility. J Insect Behav 26: 679–689.

[42] Contreras-Garduño J, Lanz-Mendoza H, Córdoba-Aguilar A (2007) The expression of a sexually selected trait correlates with different immune defense components and survival in males of the American rubyspot. J Insect Physiol 53: 612–621. 17451742 10.1016/j.jinsphys.2007.03.003

[43] Liu Z, Williamson MS, Lansdell SJ, Denholm I, Han Z, Millar NS (2005) A nicotinic acetylcholine receptor mutation conferring target-site resistance to imidacloprid in *Nilaparvata lugens* brown planthopper. PNAS 102:8420–8425. 15937112 10.1073/pnas.0502901102 PMC1150837

[44] Ahsaei SM, Tabadkani SM, Hosseininaveh V, Allahyari H, Bigham M (2013) Differential accumulation of energy by the colour morphs of the pea aphid *Acyrthosiphon pisum* (Hemiptera: Aphididae) mirrors their ecological adaptations. Eur J Entomol 110: 241–245.

